# Does sleep deprivation alter functional EEG networks in children with focal epilepsy?

**DOI:** 10.3389/fnsys.2014.00067

**Published:** 2014-04-29

**Authors:** Eric van Diessen, Willem M. Otte, Kees P. J. Braun, Cornelis J. Stam, Floor E. Jansen

**Affiliations:** ^1^Department of Pediatric Neurology, Brain Center Rudolf Magnus, University Medical Center UtrechtUtrecht, Netherlands; ^2^Biomedical MR Imaging and Spectroscopy Group, Image Sciences Institute, University Medical Center UtrechtUtrecht, Netherlands; ^3^Department of Clinical Neurophysiology, VU University Medical CenterAmsterdam, Netherlands

**Keywords:** epilepsy, EEG, sleep deprivation, network analysis, graph theory, minimum spanning tree

## Abstract

Electroencephalography (EEG) recordings after sleep deprivation increase the diagnostic yield in patients suspected of epilepsy if the routine EEG remains inconclusive. Sleep deprivation is associated with increased interictal EEG abnormalities in patients with epilepsy, but the exact mechanism is unknown. In this feasibility study, we used a network analytical approach to provide novel insights into this clinical observation. The aim was to characterize the effect of sleep deprivation on the interictal functional network organization using a unique dataset of paired routine and sleep deprivation recordings in patients and controls. We included 21 children referred to the first seizure clinic of our center with suspected new onset focal epilepsy in whom a routine interictal and a sleep deprivation EEG (SD-EEG) were performed. Seventeen children, in whom the diagnosis of epilepsy was excluded, served as controls. For both time points weighted functional networks were constructed based on interictal artifact free time-series. Routine and sleep deprivation networks were characterized at different frequency bands using minimum spanning tree (MST) measures (leaf number and diameter) and classical measures of integration (path length) and segregation (clustering coefficient). A significant interaction was found for leaf number and diameter between patients and controls after sleep deprivation: patients showed a shift toward a more path-like MST network whereas controls showed a shift toward a more star-like MST network. This shift in network organization after sleep deprivation in patients is in accordance with previous studies showing a more regular network organization in the ictal state and might relate to the increased epileptiform abnormalities found in patients after sleep deprivation. Larger studies are needed to verify these results. Finally, MST measures were more sensitive in detecting network changes as compared to the classical measures of integration and segregation.

## Introduction

An interictal electroencephalogram (EEG) is routinely acquired in patients suspected of epilepsy to support the clinical diagnosis. Nevertheless, the interictal EEG recording often lacks epileptiform abnormalities or is insufficient to determine classification of the epilepsy syndrome. EEG recordings after sleep deprivation (SD-EEG) improve the diagnostic yield of interictal EEG recordings (Malow, 2004; Smith, 2005; Wirrell, 2010). In the past, debate has centered on the question whether it is the recording of sleep itself or sleep deprivation that promotes the increased presence of epileptiform abnormalities and thereby its sensitivity. Converging evidence exists that SD-EEG recordings improve the detection of epileptiform abnormalities and help to determine classification of the epilepsy syndrome, independently of the presence of sleep during the EEG recording (Ellingson et al., 1984; Fountain et al., 1998).

The mechanism for the increased presence of epileptiform abnormalities after sleep deprivation, however, remains unclear. There is converging evidence that sleep and sleep deprivation causes variability in cortical excitability that might be related to the increased presence of epileptiform abnormalities, for review see (Badawy et al., 2012). Another, possible complementary, explanation for this clinical phenomenon might originate from an altered functional network organization after sleep deprivation (Koenis et al., 2013). Brain functioning is increasingly perceived as a complex network of interacting brain regions (Bassett and Bullmore, 2009; Bullmore and Sporns, 2009; Stam and Van Straaten, 2012), which can be studied using, among others, EEG recordings. Quantification of connections between brain regions enables characterization of functional network organization by means of network analysis. Network analysis reduces complex systems, such as the brain, to a collection of nodes (brain regions) and edges (connections between brain areas). From these networks several measures can be inferred to characterize global changes and efficiency in network organization: the clustering coefficient (a measure of segregation) and path length (a measure of integration). Normal brain functioning relies on an adequate balance of local segregation and global integration (Bullmore and Sporns, 2009; Stam, 2010). Koenis et al. showed that network organization changes in healthy subjects after sleep deprivation toward a more integrated and less segregated network (Koenis et al., 2013). Interestingly, functional networks in epilepsy are repeatedly identified as “less efficient” as this organization changes in the ictal state toward a more segregated and less integrated network (Van Diessen et al., 2013). We hypothesized that sleep deprivation would change the interictal network toward a more segregated and less integrated network, deviant from healthy controls.

Network analytical studies require various choices and assumptions that influence the outcome (Van Wijk et al., 2010). For example, it remains unclear how differences in network density (i.e., number of connections) should be taken into account when comparing networks between groups. A solution for this problem might be offered by the so-called minimum spanning tree (MST) approach. The MST approach creates a unique network based on the weighted connections between nodes. Furthermore, it connects all the nodes in the network without forming cycles and thereby reducing the connection costs. In this way, networks are obtained with an identical number of connections and thereby facilitating comparison between groups by taking only the most important connections into account. An increasing number of neuropsychiatric studies have already successfully used this approach to characterize networks and to improve group comparisons (Lee et al., 2006; Ortega et al., 2008; Schoen et al., 2011; Boersma et al., 2013). To investigate an added value of the MST approach in understanding the increased presence of epileptogenic abnormalities after sleep deprivation, we compared the results of traditional weighted network measures (clustering coefficient and path length) with MST network measures.

## Materials and methods

### Patients

Children who visited the outpatient First Seizure Clinic of the University Medical Center Utrecht, the Netherlands, between January 2006 and December 2012 were eligible for our study. We included children in whom both a routine EEG and SD-EEG recording was performed for clinical reasons. We accepted a maximum interval of 9 months between routine EEG and SD-EEG since aging is associated with alterations in functional network organization (Boersma et al., 2011). Children in whom the diagnosis of focal epilepsy was definite—as judged by the occurrence of multiple seizures, their semiology, and supported by neurophysiological recordings or neuroimaging—were compared to a control group. The control group consisted of children who were referred with clinical events suspected of seizures but in whom the diagnosis of epilepsy was eventually excluded, based on expert opinion, on clinical follow-up, and on the results of ancillary investigations, including routine and SD-EEG recordings. For both groups, we excluded children with neurological or psychiatric co-morbidities, including the presence of developmental delay of unknown origin. The institutional ethical committee approved the study and concluded that the Dutch Medical Research Involving Human Subjects Act did not apply, and written informed consent was not required.

### Data acquisition and selection

The EEG was recorded at 21 scalp electrodes (Fp1, Fp2, F8, F4, Fz, F3, F7, A2, T8, C4, Cz, C3, T7, A1, P8, P4, Pz, P3, P7, O1, and O2), according to the international 10-20 system (SystemPlus Evolution, Micromed), against G2 as a reference electrode (placed between Cz and Fz) and referenced to an average montage for further analysis. Impedance of each electrode was kept below 5 kΩ. Data was high- and low-pass filtered at 0.5 and 70 Hz, respectively. The sampling frequency was 512 Hz.

All EEG recordings were acquired for clinical purposes. Therefore, the recorded segments suitable for selection of resting state EEG epochs, were limited in their length to 2–3 min. During these segment recordings children were awake and had their eyes-closed. From these we selected four epochs of 8.19 s (each containing 4096 samples). Epochs were visually inspected by one of the authors [EvD]. The minimal number of epochs was based on a previous network study that acquired stable network characteristics within patients using four epochs of similar length (Douw et al., 2013). In order to investigate the actual contribution of sleep deprivation on functional networks, the epochs from SD-EEG recordings were selected before sleep and drowsiness, when an occipital alpha rhythm was still present and slow eye-movements were absent. Epochs were chosen free of epileptiform abnormalities, abnormal slowing and electrocardiographic or motion-induced artifacts, for which two frontoparietal and basal temporal electrodes (Fp1, Fp2, A1, and A2) were excluded. Furthermore, all epochs were selected at the beginning of the EEG recordings from EEG recordings that were conducted in the morning to increase homogeneity of our data. The epochs were independently re-inspected by a clinical epileptologist [Floor E. Jansen] on artifacts and epileptiform abnormalities. Finally, the selected EEG epochs were converted to ASCII files for further analysis.

## Evaluation of added clinical value sleep deprivation EEG

EEG recordings after sleep deprivation were performed to improve the diagnostic yield. We considered an SD-EEG of added clinical value when the SD-EEG recording provided: (1) new information regarding localization of the epileptogenic focus and (2) additional information regarding spreading of epileptiform activity. A confirmation of the clinical findings of the first EEG recording was not considered as added value.

### Functional connectivity

Communication between brain areas in EEG networks can be quantified using functional connectivity. The level to which different brain areas are functionally connected depends on the level of synchronous temporal activity, irrespective of signal amplitude (Varela et al., 2001). Although computational differences exist between various functional connectivity measures (Pereda et al., 2005), all of these measures assume that an increase in linear and nonlinear correlations of frequency specific neurophysiologic activity between two brain areas—also referred to as “synchronization”—is associated with an increased communication between both brain areas (Bassett and Bullmore, 2009; Bullmore and Sporns, 2009; Stam and Van Straaten, 2012).

In this study, functional connectivity between electrodes was computed from each selected epoch by means of the Phase Lag Index (PLI) (Stam et al., 2007) [Brainwave software; http://home.kpn.nl/stam7883/brainwave.html version 0.9.116 authored by (Cornelis J. Stam)]. A PLI value for each pair of electrodes was computed for each epoch and the average PLI (over four epochs) was used for further analysis. The PLI was calculated separately for the following frequency bands: delta (0.5–4 Hz), theta (4–8 Hz), alpha (8–13 Hz), and beta (13–30 Hz). The PLI is a measure that is less sensitive for confounding than other functional connectivity measures and quantifies the phase coupling between two time series as a value between 0 and 1 (Stam et al., 2007). The synchronization between time series is based on the consistency of the nonzero phase lag of one time series with the other. To compute the instantaneous phase difference for each time sample we used the analytical signal concept and the Hilbert transform. During the calculation the influence of volume conduction is diminished by disregarding phase differences of zero. A PLI value of 0 indicates no phase coupling between signals, or coupling with a phase difference centered on 0 ± π radians. A PLI > 0 indicates the presence of phase coupling. An index of the asymmetry of the phase difference distribution can be obtained from a time series of phase differences ΔΦ(*t*_*k*_) in the following way: *PLI* = |〈*sign*[ΔΦ(*t*_*k*_)]〉| where ΔΦ is the difference between instantaneous phases for two time-series [−π, π], *t*_*k*_ are discrete steps, and in which < > denotes the average over time *t*. A more detailed description of the calculation and specifications of PLI can be found elsewhere (Stam et al., 2007).

### Network analytical measures

#### Network construction

For each dataset, we constructed a weighted undirected network, described by the graph *G* = (*N, W*), where *N* is the set of all 17 EEG electrodes and *W* = {*w*_*ij*_} is the *N* × *N* symmetric weight matrix, where *w*_*ii*_ = 0 and *w*_*ij*_ the PLI determined between node *i* and *j*. Global network properties were quantified via weighted clustering coefficient and path length. We repeated the construction of functional networks and analysis for different frequency ranges.

#### Weighted clustering coefficient

The clustering coefficient is a measure of degree to which nodes in a graph tend to cluster together. We used the weighted clustering coefficient C as described in Grindrod (2002), Higham et al. (2007), Stam et al. (2009). To calculate C from weighted networks, the weights between node *i* and other nodes *j* should be symmetrical (*w*_*ij*_ = *w*_*ji*_) and 0 ≤ *w*_*ij*_ = 1. These conditions are fulfilled when using PLI as weight definition. The (weighted) C of node *i* is then defined as

$$C_{i}^{w}=\frac{\sum_{k\;\neq \;i}\sum_{\begin{array}{c} l\;\neq \;i\\ l\;\neq \;k \end{array}}w_{ik}w_{il}w_{kl}}{\sum_{k\;\neq \;i}\sum_{\begin{array}{c} l\;\neq \;i\\ l\;\neq \;k \end{array}}w_{ik}w_{il}}$$

in the sums of this formula *i* = *k, i* = *l*, and *k* = *l* are not included. In the case in which *w*_*ij*_ equals either 0 or 1 the definition was equivalent to the definition for unweighted graphs (Watts and Strogatz, 1998). The mean weighted clustering coefficient was defined as

$$C_{mean}^{w}=\frac{1}{N}\sum_{i\;=\;1}^{N}C_{i}^{w}$$

#### Weighted path length

for a given node *i* in the graph, the path algorithm finds the path with lowest cost (i.e., the shortest path length) between that node and every other node. For the weighted path length, the path between two nodes *i* and *j* is found by minimizing the sum of weights assigned to the edges on their path. The average path length (L) for node *i* to all other nodes is defined as

$$L_{i}^{w}=\frac{1}{N-1}\sum_{i\;\neq \;j}^{N}\min\left\{l_{ij}^{w}\right\}$$

Here, min {*l*^*w*^_*ij*_} is the weighted path length between node *i* and *j*, calculated using Dijkstra's algorithm (Dijkstra, 1959). We considered high values of the PLI as close functional distance and low values of the synchronization index as large functional distance (i.e., *l*^*w*^_*ij*_ = 1/*w*_*ij*_). In our dataset no disconnected nodes were present. The mean weighted path was defined as

$$L_{mean}^{w}=\frac{1}{N}\sum_{i\;=\;1}^{N}l_{i}^{w}$$

#### Normalization

for each functional dataset, path length and clustering coefficient were normalized using 50 surrogate networks as previously described in Stam et al. (2009). This number was sufficient to result in stable surrogate network properties (defined as less than 1% variability in network parameters such as path length and clustering coefficient). Normalized weighted path length was defined as *L*/〈*L*_*surrogate*_〉 and normalized clustering coefficient was defined as *C*/〈*C*_*surrogate*_〉.

### Minimum spanning tree

**Figure 1:**
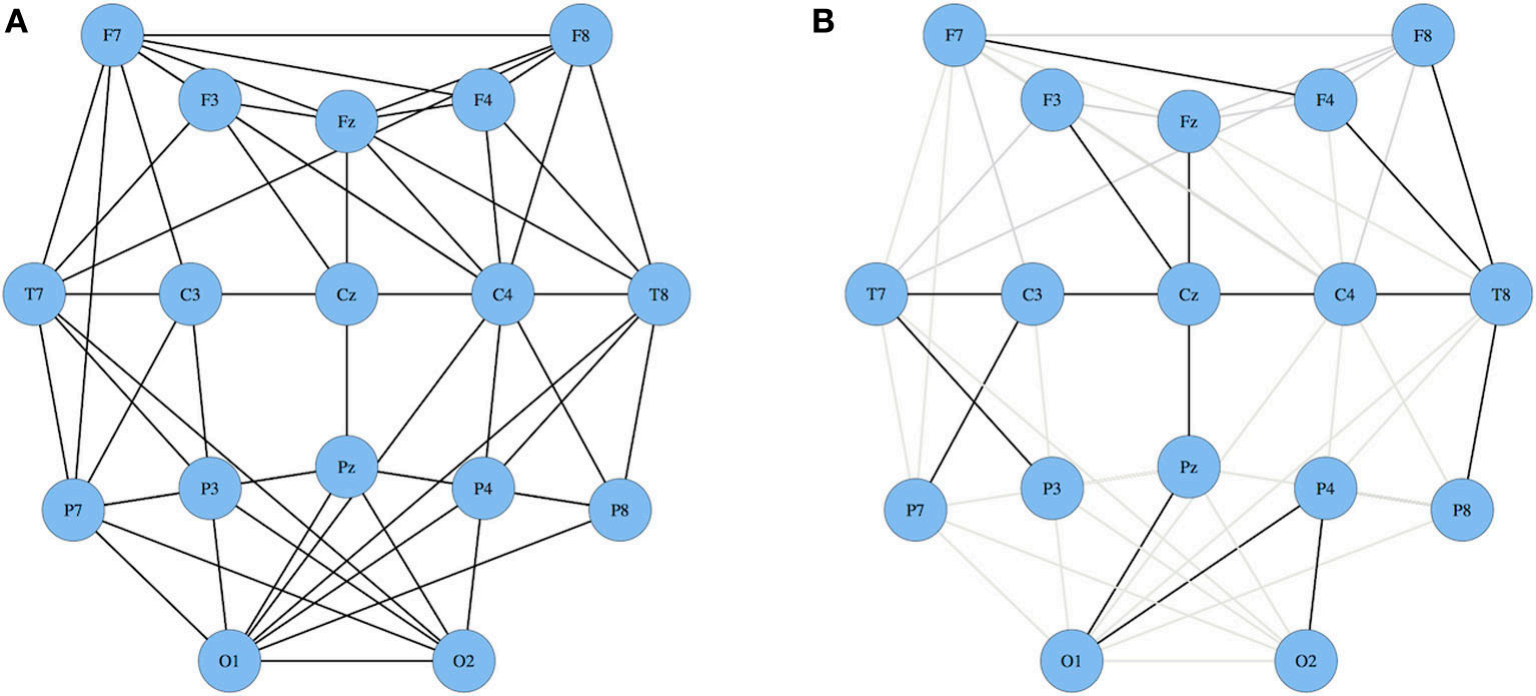
**Two schematic illustrations of networks. (A)** a standard network and **(B)** a MST network wherein all nodes are connected only once resulting in a loopless network. In panel **(B)** the black lines represent the most efficient connections in the MST network; grey lines represent the excluded functional connections.

**Figure 2:**
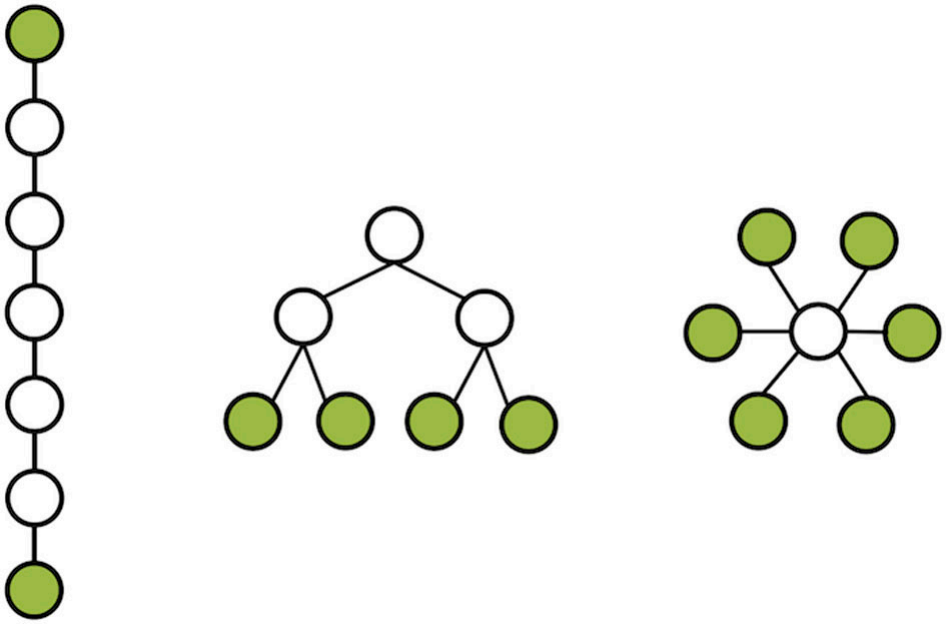
**Three network topologies based on MST network**. On the left a path-like topology with few leafs and long diameter; on the right a star-like topology with many leaves and a moderate diameter. In the middle an intermediate form combining the qualities of a line-like and star-like topology. Notify that all networks have the same number of nodes and connections. Leafs colored in green.

The MST creates a unique network based on the weighted connections between nodes. Furthermore, it connects all the nodes in the network without forming cycles and thereby reduces the connection costs. To compute the MST we used Kruskal's algorithm (Kruskal, 1956). This algorithm orders the weight of all edges in an ascending way, and subsequently starts the construction of a MST performing the following step as many times as possible: adding the edge with the highest PLI until all nodes *N* are connected in a loopless network consisting of *N* − 1 edges. Thus, if adding an edge results in formation of a cycle within the network, this edge will be skipped. Given the number of electrodes included in our analysis, trees contained 17 nodes and 16 edges. From these trees, several measures can be quantified (Boersma et al., 2013). For our study, we focused on the two most straightforward MST measures: leaf number and diameter that give information on topological features of trees (Figure 2). The leaf number is defined by the number of nodes in the tree with degree = 1 (i.e., these nodes are connected by only one edge to the network) and has a lower bound of 2 and an upper bound of m = *N* − 1. The leaf number presents an upper bound to the diameter of the spanning tree that is the largest distance between any possible pair of nodes of the tree. The upper limit of the diameter of the tree is defined as diameter =m − leaf number +2, implying that the largest possible diameter will decrease with increasing leaf number (Boersma et al., 2013). Both leaf number and diameter were normalized between 0 and 1. In summary, by excluding less important connections in the network, the MST network is built from the most efficient connections and enables a direct comparison between two networks since the number of nodes and connections are similar (Figure 1).

## Statistical analysis

First, we explored the effect of sleep deprivation in each group separately by comparing relative power spectra, network and MST measures from routine EEG and SD-EEG recordings for each frequency band with a paired *t*-test. To investigate whether a different network alteration was observed after sleep deprivation in patients compared with controls, we used a repeated measures analysis of variance (ANOVA) with type of EEG (EEG vs. SD-EEG) as within factor and group (patients vs. controls) as between factor and type of EEG × group as interaction for each frequency band. Differences between groups in age and gender were explored using independent *t*-test and chi-square test respectively. To correct for multiple comparison, we used false discovery rate correction per frequency band as each frequency band is associated with distinct network and functions (Basar et al., 2001). All analyses were performed in SPSS. A *p*-value below 0.05 was considered significant.

## Results

### Patient characteristics

In total, 21 children with a definitive diagnosis of focal epilepsy were included in this study (5 girls, mean age 10.7 ± 3.1 years). Clinical details are summarized in Table 1. Average time between EEG and SD-EEG recording was 2.4 ± 2.6 months. The control group consisted of seventeen children (7 girls, mean age 10.5 ± 2.5 years). Average time between their EEG and SD-EEG recording was 1.9 ± 1.1 months. No significant differences were found for age (*t* = −0.11, *p* = 0.9) and gender (χ^2^ = 1.3, *p* = 0.3) between patient and control groups. In three patients, anti-epileptic drug treatment was initiated after routine EEG recording and before SD-EEG recording. In 15 out of 21 patients (71%) the SD-EEG provided new information regarding localization of the epileptic focus or on the spreading of epileptiform activity and therefore considered of additional clinical value (Table 1). No epileptiform activity was found in controls during SD-EEG recording.

**Table 1:** **Patient characteristics and clinical details**.

**Patient**	**Gender**	**Age (years)**	**EEG report**	**SD-EEG report**	**Added value SD-EEG**	**Use of of AED**	**MRI findings**	**Final diagnosis**
1	F	5.1	Single SWC in right central region	Several SWCs in the right central region	Yes	Yes	No abnormalities	Cryptogenic epilepsy
2	F	6.0	Aspecific activity in the left frontal region	Several SWCs in the midparietal region. Increase of Spikes during NREM sleep	Yes	No	No abnormalities	Panayiotopoulos syndrome
3	M	7.1	No abnormalities	Several SWCs in the left frontal region while awake and during sleep	Yes	No	Increased signal intensities basal ganglia & cerebellum	Symptomatic epilepsy
4	M	7.4	Single spike midparietal	Several spikes in the midparietal region while awake and during sleep	Yes	No	Not performed	Panayiotopoulos syndrome
5	M	8.8	Active focus generating SWCs in the right centro-temporal region	Active focus generating SWCs in the right centro-temporal region while awake and during sleep	No	No	No abnormalities	Rolandic epilepsy
6	M	8.8	Spike activity in the left central region	Spike activity in the left central region	No	No	No abnormalities	Rolandic epilepsy
7	M	9.1	No abnormalities	Several spikes in the left central region	Yes	No	Small aspecific lesions cerebellum	Cryptogenic epilepsy
8	F	9.2	Slow wave activity in the left temporal region and several times paroxysmal activity	One single paroxysmal activity	No	No	No abnormalities	Cryptogenic epilepsy
9	M	9.4	No abnormalities	Epileptic activity originating from midline region	Yes	No	No abnormalities	Cryptogenic epilepsy
10	M	9.5	No abnormalities	Several spikes and sharp waves in the right hemisphere increasingly present during sleep	Yes	No	Not performed	Rolandic epilepsy
11	F	9.9	Possible spikes in the left fronto-temporal region	Several SWCs in the left temporal region spreading toward frontal region	Yes	Yes	No abnormalities	Cryptogenic epilepsy
12	M	10.2	Slow wave activity in the right parietal-central region	Several SWCs while awake and during sleep	Yes	No	Tuberous sclerosis	Symptomatic epilepsy
13	F	10.6	No abnormalities	One single sharp wave in the right hemisphere	No	No	No abnormalities	Cryptogenic epilepsy
14	M	12.2	No abnormalities	Ictal activity in the left temporo-occipital region during sleep	Yes	No	Old ischemic lesion left parietal	Symptomatic epilepsy
15	M	12.9	Aspecific abnormalities in the frontal region and isolated sharp wave complexes	During sleep increase of aspecific multifocal abnormalities	No	No	No abnormalities	Cryptogenic epilepsy
16	M	13.1	Aspecific abnormalities in the left fronto-temporal region and a single SWC in right temporal region	Several SWCs in the right central region while awake and during sleep	Yes	No	Not performed	Rolandic epilepsy
17	M	13.6	No abnormalities	Several spikes in the temporal region	Yes	No	Small lesion fronto-basal	Symptomatic epilepsy
18	M	13.8	Several SWCs in the left frontal region	Several SWCs in the left frontal region	No	No	No abnormalities	Cryptogenic epilepsy
19	M	15.0	Aspecific abnormalities in the right and left temporal region	Aspecific abnormalities one single SWC	No	Yes	No abnormalities	Cryptogenic epilepsy
20	M	15.1	Sharp wave activity in the right hemisphere	Sharp wave activity and SWCs in the right frontal region	Yes	No	No abnormalities	Cryptogenic epilepsy
21	M	16.5	Aspecific abnormalities in the left frontal region	Several spikes and SWCs in the left frontal region; during sleep generalized activity	Yes	No	No abnormalities	Cryptogenic epilepsy

F, female; M, male. SWC, Spike Wave Complex; NREM sleep, non Rapid Eye-Movement sleep; AED, anti-epileptic drugs. AED treatment started between EEG and SD-EEG recording. ^*^Final diagnosis was based on at least 1 year of follow-up, occurrence of multiple seizures, neuroimaging and neurophysiological recordings.

### Differences between regular and SD-EEG in relative power spectra per group

Paired *t*-tests were performed for relative power spectra per frequency band for patients and controls separately. In patients, no significant differences between spectra obtained from standard EEGs and SD-EEGs were found (Table 2). In controls, however, an increase of the relative spectrum in the beta frequency band was found after sleep deprivation (*p* = 0.012).

**Table 2:** **Paired *t*-test between EEG and SD-EEG recordings (for patients and controls separately) for relative power spectra in five frequency bands**.

	**Patients**					**Controls**				
	**EEG-power**	***SD***	**SD-EEG power**	***SD***	***p*-value**	**EEG-power**	***SD***	**SD-EEG power**	***SD***	***p*-value**
Delta band (0.5–4 Hz)	0.421	0.075	0.428	0.104	0.672	0.449	0.071	0.424	0.102	0.080
Theta band (4–8 Hz)	0.217	0.078	0.222	0.079	0.594	0.216	0.074	0.207	0.077	0.460
Alpha1 band (8–10 Hz)	0.167	0.089	0.160	0.095	0.585	0.154	0.081	0.157	0.107	0.791
Alpha2 band (10–13 Hz)	0.105	0.086	0.094	0.086	0.134	0.088	0.053	0.106	0.072	0.089
Beta band (13–30 Hz)	0.078	0.029	0.085	0.035	0.117	0.078	0.031	0.089	0.038	0.012^*^

Alpha band is separated in a lower and upper frequency band. SD, standard deviation.

* *Significant (p < 0.05)*.

## Differences between regular and SD-EEG in network and MST measures per group

Paired *t*-tests were performed for each network and MST characteristic per frequency band for patients and controls separately (Tables 3A,B respectively). In patients, a significant decrease in leaf number (*p* = 0.041) after sleep deprivation was found only in the alpha frequency band. In the other frequency bands, no significant differences in network measures were found after sleep deprivation. In controls, the clustering coefficient (*p* = 0.021) and leaf number (*p* = 0.014) in the delta frequency band increased after sleep deprivation and the diameter decreased (*p* = 0.037).

**Table 3A:** **Paired *t*-test between EEG and SD-EEG recordings of patients for network measures (path length and clustering coefficient) and MST measures (diameter and leaf number)**.

	**Delta band (0.5–4 Hz)**		**Theta band (4–8 Hz)**		**Alpha band (8–13 Hz)**		**Beta band (13–30 Hz)**	
	***t*-value**	***p*-value**	***t*-value**	***p*-value**	***t*-value**	***p*-value**	***t*-value**	***p*-value**
Path length	0.774	0.448	0.150	0.882	1.492	0.151	1.059	0.302
Clustering coefficient	0.317	0.755	−1.840	0.081	−1.803	0.086	1.304	0.207
Diameter	0.383	0.705	0.447	0.660	1.366	0.187	−0.923	0.367
Leaf number	1.072	0.297	−0.103	0.916	−2.189	0.041^*^	1.769	0.092

A positive t-value indicates an increase of network/MST measure after sleep deprivation. A negative t-value indicates a decrease of network/MST measure after sleep deprivation.

* *Significant (p < 0.05)*.

**Table 3B:** **Paired *t*-test between EEG and SD-EEG recordings of controls for network measures (path length and clustering coefficient) and MST measures (diameter and leaf number)**.

	**Delta band (0.5–4 Hz)**		**Theta band (4–8 Hz)**		**Alpha band (8–13 Hz)**		**Beta band (13–30 Hz)**	
	***t*-value**	***p*-value**	***t*-value**	***p*-value**	***t*-value**	***p*-value**	***t*-value**	***p*-value**
Path length	1.104	0.286	−1.283	0.218	1.043	0.313	−0.353	0.729
Clustering coefficient	2.561	0.021^**^	−0.449	0.659	0.770	0.452	−0.687	0.502
Diameter	−2.269	0.037^**^	0.438	0.668	−1.409	0.178	−0.682	0.505
Leaf number	2.759	0.014^**^	−0.502	0.623	1.007	0.329	−0.054	0.958

A positive t-value indicates an increase of network/MST measure after sleep deprivation. A negative t-value indicates a decrease of network/MST measure after sleep deprivation. ^*^Significant (p < 0.05),

** *Significant after correcting for multiple post-hoc comparisons (false discovery rate test)*.

### Interaction in network and MST measures

**Figure 3:**
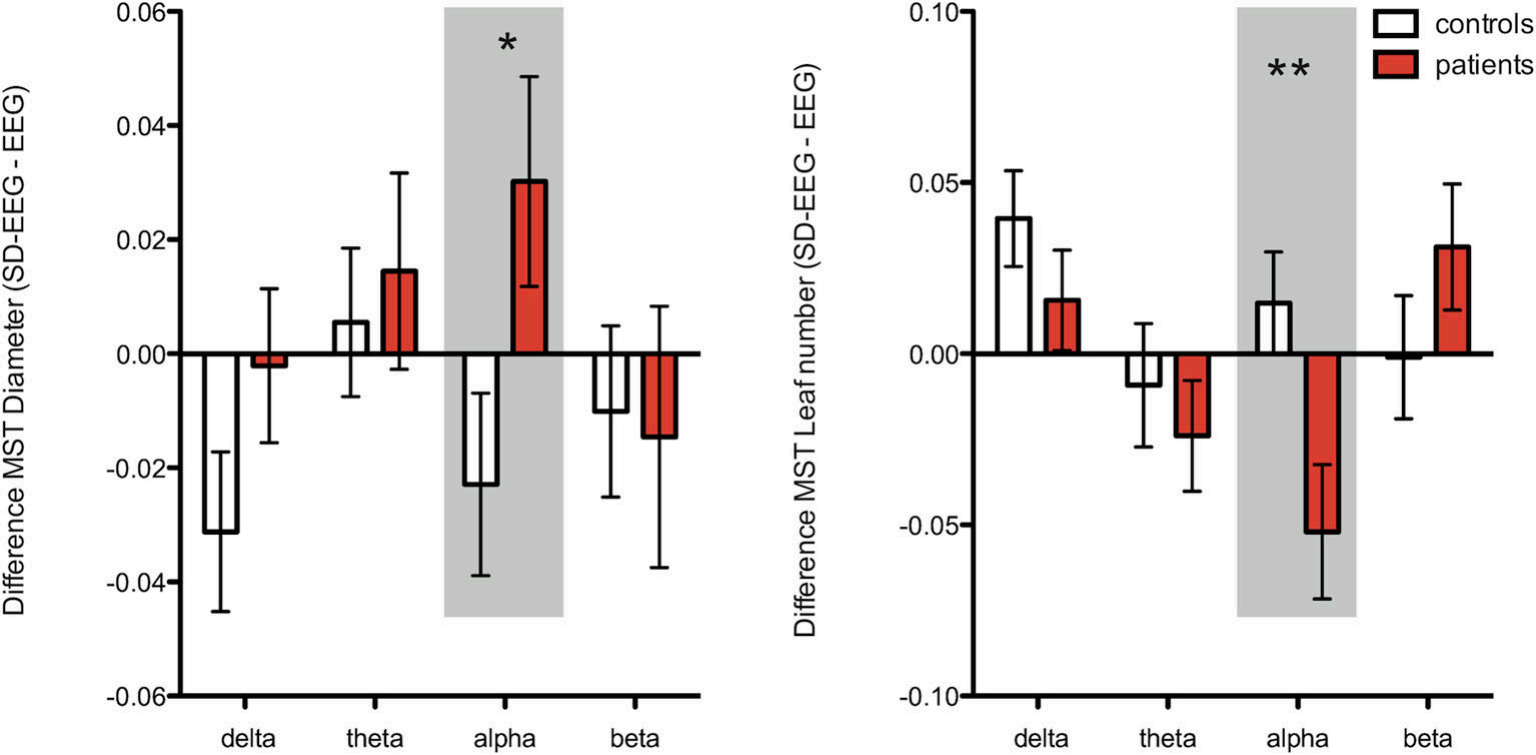
**Illustration of interaction effects from MST measures diameter (left graph) and leaf number (right graph) per frequency band as revealed with a repeated measures ANOVA (mean values and standard error of the mean bars).** In this analysis we included only patients in whom the SD-EEG was of added value (*n* = 15), and all controls (*n* = 17) (Table 4). There was a significant interaction for diameter in the alpha band; the diameter increased in patients whereas an opposite effect was found for controls. For leaf number, a significant interaction was found in the alpha band; the leaf number decreased in patients whereas an opposite effect was found for controls. Together, these results in the alpha band suggest a shift toward a path-like topology for patients after sleep deprivation and a shift toward a star-like topology for controls. ^*^Significant (*p* < 0.05), ^**^Significant after correcting for multiple *post-hoc* comparisons (false discovery rate test).

Repeated measures ANOVA were performed for each network and MST characteristic separately for each frequency band. Significant interaction was only found for the leaf number (*p* = 0.03) and a trend for diameter (*p* = 0.061) in the alpha frequency band (Table 4). The interaction for leaf number and diameter increased when including only patients in whom SD-EEG recording was considered to be of additional diagnostic value (*p* = 0.009 and *p* = 0.03 respectively) (Figure 3).

**Table 4:** **Interactions from repeated measures ANOVA for each network and MST measures**.

	**Interaction(all patients, *n* = 21)**		**Interaction (added value SD-EEG, *n* = 15)**	
	Type of EEG^*^group		Type of EEG^*^group	
	***F*-value**	***p*-value**	***F*-value**	***p*-value**
**DELTA-BAND (0.5–4 Hz)**				
Path length	0.115	0.737	0.008	0.928
Clustering coefficient	2.123	0.154	0.456	0.505
Diameter	3.976	0.054	2.256	0.144
Leaf number	2.144	0.152	1.359	0.253
**THETA-BAND (4–8 Hz)**				
Path length	1.018	0.320	1.395	0.247
Clustering coefficient	0.394	0.534	0.321	0.575
Diameter	1.558	0.220	0.183	0.672
Leaf number	0.103	0.750	0.361	0.552
**ALPHA-BAND (8–13 Hz)**				
Path length	0.075	0.785	0.059	0.809
Clustering coefficient	2.881	0.098	2.038	0.164
Diameter	3.735	0.061	4.724	0.038^*^
Leaf number	5.101	0.030^*^	7.680	0.009^**^
**BETA-BAND (13–30 Hz)**				
Path length	0.977	0.330	0.216	0.645
Clustering coefficient	1.852	0.182	0.077	0.783
Diameter	0.058	0.812	0.030	0.864
Leaf number	1.360	0.251	1.539	0.224

In the left column, the interactions when including all patients (n = 21). In the right column, the interactions when including only those patients in whom the SD-EEG was of added clinical value (n = 15).

* Significant (p < 0.05),

** *Significant after correcting for multiple post-hoc comparisons (false discovery rate test)*.

## Discussion

An EEG after sleep deprivation is often performed in patients suspected of epilepsy when the standard EEG recording is inconclusive. Little is known, however, about the mechanism behind the increased sensitivity of SD-EEG recordings in epilepsy. In this study we investigated whether a network analytical approach could clarify this phenomenon. Our repeated measures analysis for leaf number and diameter suggested an interaction between patients and controls after sleep deprivation: patients showed a shift toward a more path-like MST network whereas controls showed a shift toward a more star-like MST network (Figure 2). This shift difference was more pronounced when including only the patients in whom the SD-EEG recording was of additional clinical value (Table 4). Together with the increased presence of epileptiform abnormalities after sleep deprivation in patients, the network shift toward a more path-like MST network could possibly reflect an inadequate compensatory mechanism of the epileptic brain, although this requires to be confirmed using data from future studies with larger sample sizes.

Previous research has shown that functional networks change during seizure generation and propagation into a more regular network organization (i.e., highly segregated and poorly integrated network) (Ponten et al., 2007; Schindler et al., 2007; Kramer et al., 2008, 2010; Schindler et al., 2008; Ponten et al., 2009). This shift toward a regular topology might depend on the decreased centrality of so-called “hub-nodes” in the network or an altered synchronizability during the ictal state (Van Diessen et al., 2013). Furthermore, long-term continuous evaluation of functional networks derived from intracranial recordings, revealed large fluctuations in clustering coefficient and path length during the day (Kuhnert et al., 2010). These fluctuations over time, largely attributed to daily rhythms, showed an increased regularization of functional networks during night-time in patients with focal epilepsy (Kuhnert et al., 2010) whereas a shift toward a more optimal segregation and integration of the network during sleep was observed in healthy subjects (Ferri et al., 2008). Possibly, this shift toward a more regular network during sleep in patients with epilepsy explains why the epileptic brain is more susceptible to both epileptiform discharges and seizures during sleep. However, this remains speculative as we cannot infer a causal relation between network alteration and an increased presence of interepileptic discharges after sleep deprivation based on our results. We did not investigate network alterations during sleep, but a similar mechanism could explain the increased presence of epileptiform discharges after sleep deprivation. This study suggests that the network organization shift toward a more path-like topology in patients with epilepsy (i.e., increased diameter and decreased leaf number). Considering the path-like network as a network wherein nodes are less centrally connected and share basic characteristics with a regular network, it might be possible that the mechanisms underlying sleep deprivation-induced network alterations mimic the changes of a functional network during the ictal state or during sleep. Interestingly, these changes in network organization could coincide with an increase of cortical excitability that has previously been described as a pro-convulsive effect, accountable for the induction of epileptogenic activity after sleep deprivation (Badawy et al., 2006; Manganotti et al., 2006). As mentioned previously, functional connectivity in EEG networks is based on the assumption that an increased synchronization of frequency specific neurophysiologic activity implies an improved communication between brain areas. The change toward a more path-like network in patients with focal epilepsy after sleep deprivation suggests an increased synchronization of spatially closely related brain areas. The underlying mechanism behind the increased synchronization could indeed be an increased cortical excitability, as repeatedly shown by studies combining high-frequency transcranial magnetic stimulation and EEG connectivity measures in healthy controls, particularly in the alpha frequency band (Fuggetta et al., 2008; Plewnia et al., 2008). To which extent this relation is also accountable for epilepsy remains speculative until further investigations will simultaneously map cortical excitability and associated changes in function network organization. Furthermore, we found the largest group difference in the alpha frequency band. Previous studies have suggested that each frequency band is associated with distinct networks and cognitive processes (Von Stein and Sarnthein, 2000; Basar et al., 2001). For example, spontaneous alpha activity is typically found when eyes are closed whereas task-related alpha activity is typically found during sensory, motor and primarily top-down cognitive processes (Von Stein and Sarnthein, 2000). Although it remains uncertain how our results relate to changes in the cognitive domain, they suggest an involvement of the alpha frequency band in normal sleep physiology. However, the exact relationship remains an open question.

The MST approach has been suggested as an appropriate method to overcome certain limitations of network studies, particularly in relation to differences in network densities between groups (Van Wijk et al., 2010). Nevertheless, few studies have compared standard network measures, such as clustering coefficient and path length, to MST measures (Boersma et al., 2013). We found particular network alteration between controls and patients for the MST measures leaf number and diameter, suggesting that these measures are perhaps more sensitive to network alterations in patients with epilepsy. Larger studies investigating and comparing both approaches are needed to verify this. In addition, like clustering coefficient and path length, leaf number and diameter are highly correlated, arguing for more and distinct MST features to characterize networks.

To date, only one study has investigated the influence of sleep deprivation on functional networks (Koenis et al., 2013), but only in healthy subjects. Koenis and others reported network alterations in the alpha frequency band during an eyes-closed condition, namely a shift toward a more random network. In our control group, we found an increase in clustering coefficient, a decreased diameter and increased leaf number in the delta frequency band. Together, these results suggest a more star-like MST network in controls after sleep deprivation. Interestingly, a star-like MST network shares basic characteristics with that of a random network organization (i.e., a poorly segregated and highly integrated network). Although we found a similar shift in network organization as Koenis and others, it is challenging to explain the differences in frequency bands between both studies (alpha vs. delta). Possibly, a different patient population (age) and methodology (different EEG recordings) are accountable for these differences (Van Wijk et al., 2010; Smit et al., 2012). Besides network measures, we also measured the relative power spectrum. In accordance with previous literature, we found an increase in beta power spectrum after sleep deprivation in healthy subjects (Gast et al., 2011) whereas we did not find this effect in patients. Possibly, the lack of increased beta power in patients after sleep deprivation reflects an inadequate compensation of the epileptic brain to maintain beta frequency band specific functions, such as integration of multi-sensory information (Von Stein and Sarnthein, 2000).

Although our study provides further insight in the mechanism of increased sensitivity of EEG recordings after sleep deprivation, several limitations should be mentioned. First, our patient sample is limited (21 children) and included only children with focal epilepsy. One could therefore argue that our results might be restricted to this age range and type of patients. Nevertheless, the increased sensitivity of SD-EEG recordings is particularly manifested in children suspected of focal epilepsy (Shinnar et al., 1994) and our population is therefore ideal to study this phenomenon. Secondly, the control group contained children who were initially suspected of having suffered from an epileptic seizure. Although epilepsy was excluded after clinical evaluation and follow-up, this could have introduced a bias in our results. As compared to truly healthy children who never experienced any paroxysmal event, the network organization of our control group could be altered as well. As a consequence this might have reduced our statistical group differences. Otherwise, it would be difficult to receive ethical approval for performing SD-EEG recordings in a healthy pediatric population. Furthermore, since we used clinical EEG recordings, there was only a limited fraction of EEG recording available from which we could select resting state epochs. Based on previous research, we assume that four epochs is enough to ensure stable network measures (Douw et al., 2013). The availability of more epochs would allow further exploration on the variability—and thus stability—of these network measures by, for instance, performing a leave-one-out analysis. Unfortunately, in this study we did not have sufficient data to do so, but we suggest future studies to characterize this variability using long-term EEG recordings. In three patients anti-epileptic drugs were started before the SD-EEG recording (patients 1, 11, and 19). Considering the potential influence of anti-epileptic drugs on functional networks (Van Diessen et al., 2013), this could have deviated our results. In all three patients, however, treatment started only a few days before SD-EEG recording and was therefore still at a very low dosage. An additional analysis, after excluding these patients, revealed similar results. Finally, our sample size is small and the FDR-correction to minimize inflation of the type I error might not be perfect. Additional validation is therefore needed to confirm our findings. Despite these limitations, we believe that our results legitimate a larger network study wherein the mechanism of increased sensitivity of EEG recordings after sleep deprivation will be investigated more thoroughly. To enable a subanalysis of different epilepsy syndromes and allow correlation analysis between network measures and clinical characteristics such as age, gender and use of anti-epileptic drugs a larger study cohort is required.

In conclusion, this study provides insights into the mechanisms behind the increased presence of epileptiform abnormalities after sleep deprivation in children with focal epilepsy. We suggest that an inadequate compensatory shift of the epileptic network toward a more path-like topology after sleep deprivation is accountable for the increased epileptiform abnormalities often found in patients with epilepsy.

## Author contributions

Designed the experiments: Eric van Diessen, Willem M. Otte, Kees P. J. Braun, Cornelis J. Stam and Floor E. Jansen. Performed the experiments: Eric van Diessen, Willem M. Otte. Wrote the graph analysis software: Cornelis J. Stam. Analyzed the data: Eric van Diessen, Willem M. Otte. Wrote the paper: Eric van Diessen, Willem M. Otte, Kees P. J. Braun, Cornelis J. Stam and Floor E. Jansen.

## Funding

Eric van Diessen, Willem M. Otte, and Kees P. J. Braun are supported by the Dutch National Epilepsy Fund (NEF 09-93, NEF 12-05 and NEF 08-10, respectively).

### Conflict of interest statement

The authors declare that the research was conducted in the absence of any commercial or financial relationships that could be construed as a potential conflict of interest.
